# Assessment of Need for Recovery and Its Relationship With Work Characteristics and Health in a Sample of Chinese Doctors: A Cross-Sectional Survey

**DOI:** 10.3389/fpubh.2021.600179

**Published:** 2021-05-07

**Authors:** Tao Sun, Yu Shi, Dong Yin, Shu'e Zhang, Xiaohe Wang

**Affiliations:** ^1^Department of Health Management to Medical College, Hangzhou Normal University, Hangzhou, China; ^2^Department of Health Management, College of Public Health of Harbin Medical University, Harbin, China

**Keywords:** need for recovery, cross-sectional survey, Chinese doctors, workplace well-being, self-reported health outcomes

## Abstract

**Background:** China is launching an unprecedented health care system reform. However, the long-term interests of doctors seem to have been ignored during this process especially considering that the work environment and work-life balance for doctors have severely deteriorated over the past decade.Their well-being and health are facing substantial threats due to heavy workloads and inadequate recovery opportunities. This study aimed to investigate the extent of need for recovery (NFR) among Chinese doctors and to examine their work-related fatigue. The study also examines the relationship of NFR with workplace satisfaction and health outcomes among Chinese doctors.

**Methods:** A total of 2,617 doctors from 30 administrative regions in China participated in this study to assess the need for recovery and its relationship with work characteristics and health. A cross-sectional survey was conducted using the Chinese version of the Need for Recovery Scale (NFRS). Participants were invited to complete an anonymous online survey during May 2016. Data were analyzed using descriptive statistics, one-way ANOVA, reliability analysis, Pearson correlations, and hierarchical multiple regression analysis.

**Results:** Significant differences in NFR scores were found across demographic characteristics such as age, service years, hospital levels, educational attainment, professional positions, work shifts, and working time. Regardless of any illnesses they might be experiencing, about 70.0% of participants remained at their job even though many doctors (22.1%) must continue working under the policies of the organization, which led to more pronounced NFR (*P* < 0.001). Further, a higher NFR was negatively related to workplace well-being and self-reported health outcomes of participants.

**Conclusions:** Work-induced fatigue is a growing threat to doctors in China and their recovery opportunities are extremely limited in the workplace. High NFR exerts a considerable effect on their workplace well-being and health. China's hospital managers should pay close attention to the fact that doctors have little chance of recovery, and should offer doctors' positive encouragement and support to enhance well-being. To improve doctors' working conditions, targeted prevention policies must be introduced by policymakers to control this spreading crisis.

## Background

Over the past decade, China has undertaken a series of reforms to promote an equitable and efficient health care delivery system (1). Despite numerous achievements that have been realized to some extent, a number of critical issues have still not been solved (2), and new problems and dilemmas are constantly emerging. Violence against healthcare staff in hospitals has become a prevalent public health problem (3) and has attracted considerable attention from citizens, the media, scholars, and China's government (4). As a result, how to deal with the doctor-patient relationship is raising a new and additional source of stress among doctors in China. As a consequence, working conditions and work-life balance among Chinese doctors continues to deteriorate (5).

China's government declares that their reforms follow a principle that is referred to as a “Person-Centered Framework”; however, most of the policies developed in the area of healthcare reform during recent years seem to only exhibit a patient-centered approach. Further, the various policies aimed at reducing the cost of healthcare services and enhancing the quality of health services are continually adding increased pressure on public hospitals. In order to comply with the policy goals, Chinese doctors are suffering from diverse challenges; increased workload without increased revenue in the public hospital setting has caused more complaints and burnout among physicians. The occupational health and wellness of Chinese doctors escape the notice of policy-makers notice all the time, which, in turn, results in a vicious circle: if the participation of doctors is absent,any series of reforms to China's healthcare system cannot proceed smoothly (6).

Recently, Kelly et al. proposed an Employee-Centered Care Model and suggested that the promotion of healthcare providers' health and wellness contributed to enhancing benefits in the areas of health care costs, access, and patient satisfaction (7). In reality, however, the workload and fatigue of doctors has received little attention (8) in China. In short, a focus on doctors' working environments, occupational health and safety, and labor rights, as an important component of the healthcare system, should be emphasized in healthcare reforms.

During workdays, individual physical and mental resources are often used and depleted to meet work-related requirements (9). Work-induced fatigue is a major topic in the domain of occupational health (10) and is a common experience for healthcare staff (11), especially for doctors, who work under an organizational environment characterized by long working hours, shift work, frequent night duty, heavy workloads, high work stress, inadequate sleep, and short breaks between shifts (3). In addition, doctors need to engage in emotional labor strategies, as a means of displaying organizationally appropriate emotions in the hospital setting including deep acting, surface acting, and the suppression of naturally felt emotions (12), in order to create close relationships with their patients and patients' relatives, which will increase their fatigue-related risks. These issues are especially problematic in urban public hospitals in China due to increasing medical service demands and an insufficient number of doctors (2). A large number of patients force Chinese doctors into hectic work schedules (13), in which their role is to promote patients' wellness, rather than their own (14). Accordingly, it is a common and inevitable outcome that, along with depletion of psychological resources that can counter negative psychological states such as depression, anxiety, and other psychiatric or medical disorders (15), Chinese doctors frequently suffer from prevalent fatigue (16). One issue for Chinese policymakers is how to help doctors maintain work-recovery balance and avoid burnout while promoting excellent patient care (14).

Need for recovery (NFR) is one topic regarding the work-related fatigue issue that has attracted considerable attention from both scholars and practitioners. NFR refers to a variant of the fatigue experience (17). It is regarded as an early diagnosis of chronic fatigue and is a breakthrough point in the prevention of chronic fatigue (17). Thus, as an early sign of employees' prolonged fatigue and an indication of the need to take a break and recuperate from work demands (18), NFR is frequently investigated in various fields such as sociology, organizational psychology, and occupational health psychology (19). Previous studies have demonstrated that NFR is reflected in subjective evaluations of short-term effects by self-reported statements regarding a working day (20). It is induced when employees lack time to recover during periods of work (21). NFR can also be identified as an indicator of failing to recover from the effects of fatigue, which is similar to an initial stage of a continuous process of fatigue (17).

Numerous studies have investigated the causes of NFR, which mainly include work characteristics (22) (e.g., pace and amount of work, physical effort, skill utilization, task autonomy, relationship with colleagues, relationship with direct supervisor, and job security), work-family conflict (23), job stressors (24), low quantity of sleep/rest (25), and poor psychological detachment (24). In short, due to an imbalance between job demands and available resources, both elevated job demands (26) and reduced job control and resources (support) (27) show a robust prognostic value for the level of NFR.

It is reasonable and understandable that hospital managers encourage their doctors to work diligently, so as to improve the efficiency and quantity of the doctor's service, especially in these times characterized by a shortage of human resources in the healthcare system. However, it has been shown that the effect of job involvement is a double edged sword for the staff (28), considering that doctors' time and energy is limited. Similar to “recharging the batteries,” recovery is indispensable for all doctors because it enables them to meet and be ready for new challenges. Their energy and attention both need to be restored, and suitable rest and relaxation are required for fatigued doctors. Moreover, higher levels of job involvement will consume their physical strength and mental resources under continuously uninterrupted work duties, resulting in inadequate recovery opportunities in the hospital workplace. If high NFR can't be relieved, NFR may be an indicator of well-being, health, safety, and performance problems. Although these are serious and realistic problems, they have not been the subject of extensive academic study to this point. The present research is the first to examine the subject of NFR among Chinese doctors, associated factors, and its effects.

## Research Hypotheses

Previous studies have indicated that accumulated NFR contributes to more serious costs in terms of “load effects” (29), which in turn led to a collection of emotional, cognitive, and behavioral symptoms characterized by motivational deficits, feelings of work overload, social withdrawal, irritability, subjective health complaints (26), lack of energy for new efforts, and reduced performance. Further, if a doctor's NFR is not addressed, sleep deprivation and overfatigue are likely to occur, which in turn can lead to greater risk of clinical burnout (30). In summary, if the NFR is not adequately addressed it can have very negative consequences in many areas of doctors' lives. Thus, the following hypotheses are proposed in this study conducted with a group of Chinese doctors.

### Hypothesis 1: There Is a Negative Association Between NFR and Life Satisfaction of Doctors

High workload is related to poor psychological detachment and the ability to relax during non-work time, which in turn hinders daily recovery for doctors (24). Thus, the time for leisure activities and social activities are sharply reduced for doctors. As a consequence, a prolonged activation of negative affect related to clinical work will be induced (31). Moreover, a doctor who does not have the opportunity for adequate recovery may have poor psychological detachment and the inability to refrain from job-related thoughts, which results in an increasing risk of emotional exhaustion (32). Thus, NFR can result in doctors maintaining a state of prolonged activation when at home or during leisure time, which is then associated with thinking and ruminating about work-related issues (33). This suggests that the doctor may feel overwhelmed and will often result in a situation where they continue to think about clinical duties to be accomplished even when they are at home or during leisure time. This may lead to a situation in which doctors' energy is constantly consumed which in turn increases their psychological stress. This leads to our second hypothesis:

### Hypothesis 2: NFR Is a Positive Predictor of Psychological Stress Among Doctors

Recovery experiences can protect and foster personal resources that are necessary to achieving work goals, may reduce job demands and the associated physiological and psychological costs, and motivate growth, learning, and development among doctors (31). Thus, adequate recovery assists in meeting the basic psychological needs of autonomy, belongingness, and competence, which are important job resources for doctors; meeting these can also be intrinsically motivating for them. In addition, recovery experiences engender a feeling of mastery; doctors with an adequate recovery are likely to perceive having greater control over leisure time (34), which can help them to address new challenges or learn new things during leisure time. As a consequence, recovery experiences can foster a positive satisfaction regarding the aspect of doctors' career evaluation. Conversely, high levels of NFR suggest that the doctor doesn't devote enough time to continuously improve on their own and the sense of their development and competence may be limited. Therefore, the following hypothesis is proposed in this study:

### Hypothesis 3: NFR Reduces Doctors' Sense of Career Success

A misalignment between the internal circadian rhythm and the work schedule has been found to be a crucial cause of sleep disorder (35). Due to long working hours and overtime, doctors with lack of recovery opportunities have insufficient breaks. In this case, NFR can trigger disturbances of biological and social circadian rhythms (36). High NFR as a sign of occupationally-induced fatigue is prone to lead to a vicious circle, in which doctors might give extra effort at the beginning of each working period to rebalance the suboptimal state and to avoid faulty operation (37). As a result, doctors who have insufficient time to recover remain vulnerable to sleep loss. Thus, the following hypothesis is proposed in this study:

### Hypothesis 4: NFR Is Related to Poor Subjective Sleep Quality

Numerous studies have demonstrated that people with long-term inadequate recovery during non-work time are more likely to experience a series of risks of physiological problems such as cardiovascular diseases (38), neuroendocrine reactivity (20), physical symptoms (39), muscular tension (40), and musculoskeletal problems (41). Indeed, the hazards caused by NFR have been widely studied and the relationship between NFR and health appears to be quite robust. However, even though a large number of Chinese doctors are suffering from overload, their high NFR and its associated impact get very little attention. Therefore, the following hypothesis is proposed in this study:

### Hypothesis 5: NFR Is a Positive Predictor of Low Levels of Self-Reported Health Among Doctors

**Figure 1:**
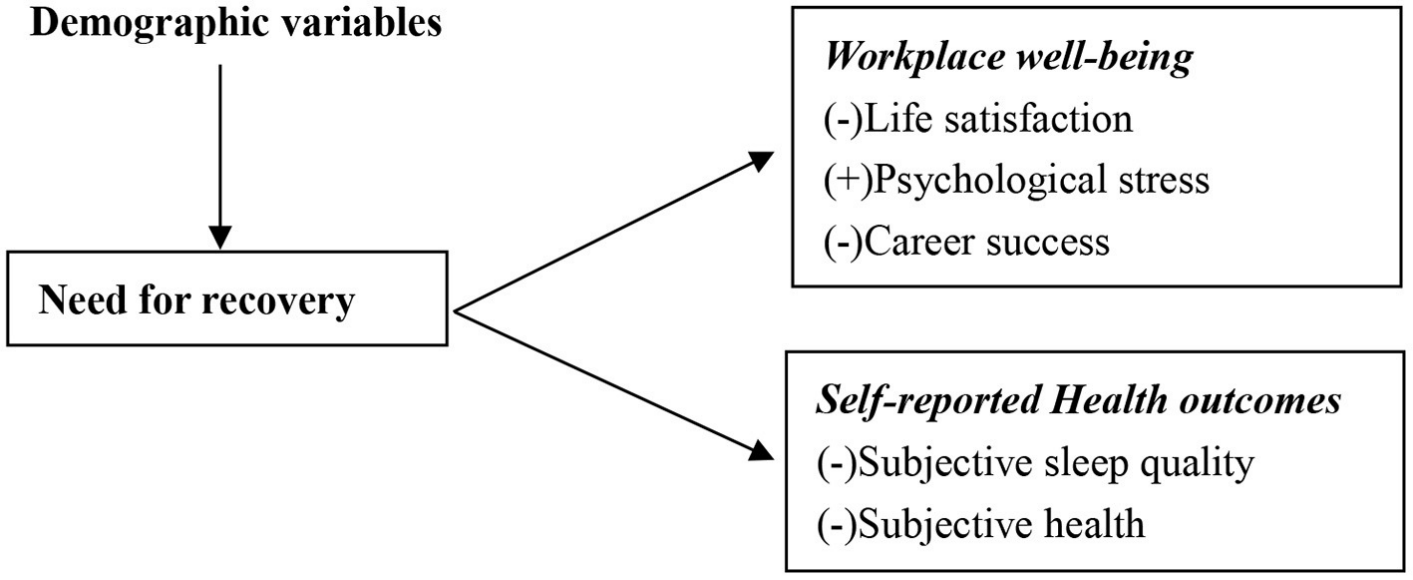
The conceptual framework of study.

In summary, the purpose of present study is to gain further insight on the status of NFR among Chinese doctors and associated factors, and to clarify the relationship between NFR, workplace well-being and self-reported health outcomes of Chinese doctors. A conceptual framework of the study is provided in Figure 1.

## Methods

### Participants and Procedures

**Figure 2:**
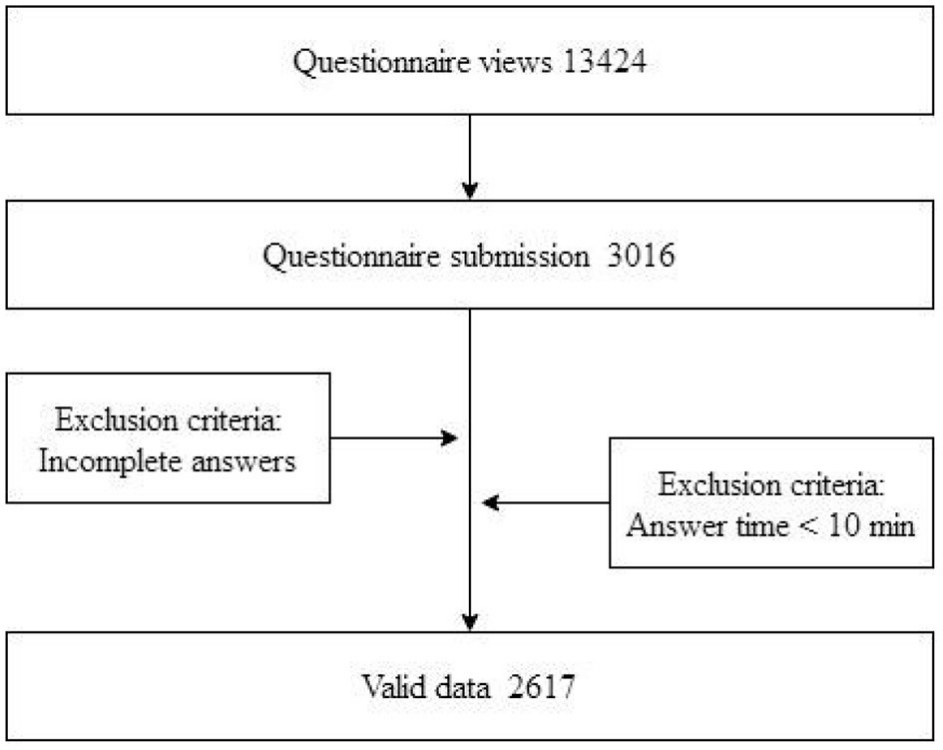
Flowchart of data acquisition.

An anonymous online cross-sectional survey was completed by doctors across the country during May 2016 in 30 of the 34 Chinese administrative regions. Participants did not include doctors from Tibet, Taiwan, Hong Kong, and Macao (Macau). Approximately 50 doctors from the authors' unit were recruited as original respondents to our survey, while the other participants were colleagues or classmates invited by the original survey respondents. The authors' unit is a well-known medical college which has a great number of graduates who have worked throughout the country and have formed a vast alumni network which could be accessed to assist in data collection. Thus, a “snowball” sampling method (word of mouth) using the alumni network was employed. Since the social media platform WeChat has been widely used in China, it was very easy to send a website link (https://www.wjx.cn/) about our survey to the mobile phones of potential participants. This link was sent to 13,424 people, and 3,016 questionnaires have been submitted successfully. A total of 2,617 valid questionnaires have been obtained, excluding incomplete answers and questionnaires that take <10 min to answer. The effective recovery rate of the questionnaire was 86.8%. The specific data acquisition process is shown in Figure 2.

The research described in the present article meets the ethical guidelines of the ethics committee of the College of Public Health, Harbin Medical University and was approved by the Ethics Committee of the Harbin Medical University (ECHMU). Written informed consent could not be obtained because of the anonymous survey approach; hence, oral informed consent for the survey was approved by the ECHMU and obtained from each doctor. Before distributing the questionnaire, we informed participants of the anonymity and privacy protection measures of the questionnaire. Dialogue and alert pop up boxes were used to remind participants who agreed to complete the survey to send their replies to our research group. Thus, once a questionnaire was completed, it was assumed that the doctor had verbally agreed to participate in our survey by reference to the Wen's criteria (3).

### Measures Need for Recovery

The NFR was assessed by using The Need for Recovery Scale (NFRS) which includes 11 items developed by Sluiter (37); the Chinese version of NFRS was translated by Qi Xin-liang (42). The items of the NFRS measure symptoms with yes/no questions to indicate the short-term effects of a day of work; thus, each item is dichotomously scored (no = 0, yes = 1). The total NFRS score is calculated by summing up the scores of the 11 constituent dichotomous items, resulting in a score ranging from 0 to 11, with higher scores indicating a higher degree of NFR. The Cronbach's alpha for the scale was 0.850.

### Workplace Well-Being

Three variables including life satisfaction, psychological stress, and subjective career success were selected to represent the workplace well-being of doctors. With reference to previous research (43), life satisfaction (44), psychological stress (45), and subjective career success (46) were each measured using one item. Items were rated on five-point Likert scales: life satisfaction–1 = extremely unsatisfied, 5 = quite satisfied; psychological stress–1 = not at all, 5 = very much; subjective career success–1 = least successful, 5 = most successful.

### Self-Reported Health Outcomes

Two single items were used to measure doctors' self-reported health outcomes. Based on research by Fein and Skinner (47), overall subjective health was estimated by a widely used single-item measure (“In general would you say your health is”: 4 = excellent, 3 = very good, 2 = good, 1 = poor). Subjective sleep quality (48) was measured by the question “How would you evaluate last night's sleep?”; the response format ranged from 1 = very bad to 4 = very good.

In addition, this study also collected demographic information from doctors including gender, age, service years, hospital level, marital status, and educational attainment. Further, the following occupational factors were considered: hours of work, shift work, psychological detachment from work during non-work time, and continuously working while suffering from illness. A single-item question was provided to participants to assess psychological detachment during non-work time: “Do you still need to think about work when you are resting?”; the response format ranged from 5 = No detachment (I must pay close attention to my responsibilities in my department) to 1 = Overall detachment (I don't need to care about my responsibilities in my department at all).

### Statistical Analysis

The NFR score were examined using one-way ANOVA. Pearson's correlation coefficients were used to estimate correlations between the NFR, workplace well-being, and self-reported health outcomes. Hierarchical linear regression analysis was performed to test the effects of groups of independent variables on workplace well-being and self-reported health outcomes. The demographic variables related to NFR in univariate analyses (*P* < 0.05) were entered into step 1 of the hierarchical regression analysis model, to eliminate their interference on the dependent variables. We also addressed the relative impact of each category of all variables on workplace well-being and self-reported health outcomes by this analysis model. In the step 2, both the demographic variables and NFR were entered into the model to test NFR's explained variance in the dependent variables. We provided data including *F*, and Δ*R*^2^. Standardized regression coefficient (β) and *P*-value for each step in the regression model. In this study, statistical significance was set at *P* < 0.05 (two-tailed). All of the analyses were conducted using SPSS 24.0 (SPSS, Inc., Chicago, IL, USA) for Windows.

## Results

### Demographic Information

Demographic variables of participants and the ANOVA models for the NFR of Chinese doctors are shown in Table 1.

**Table 1:** Characteristics of the respondents (*n* = 2,617).

**Characteristics**	**N**	**%**	**Median(Q1,Q3)**	***F***	***P***
**Overall level**					
NFR scores	2,540	97.06	9.00(7.00-10.00)		
Missing value	77	2.94			
**Age**				9.69	*P* < 0.001
≤ 30	587	22.43	9.00 (6.00-10.00)		
31-40	1,224	46.77	9.00 (7.75-10.25)		
41-50	658	25.14	9.00 (6.00-10.00)		
≥51	119	4.55	8.00 (6.00-10.00)		
Missing value	29	1.11			
**Service Years**				10.28	*P* < 0.001
≤ 10	1,088	41.57	9.00 (7.00-10.00)		
11-20	720	27.51	10.00 (7.00-11.00)		
≥30	376	14.37	9.00 (6.00-10.00)		
Missing value	433	16.55			
**Hospital level**				10.85	*P* < 0.001
Large-scale general hospital	1,740	66.49	9.00 (7.00-10.00)		
General hospital	733	28.01	9.00 (6.00-10.00)		
Primary hospital	139	5.31	9.00 (5.00-10.00)		
Missing value	5	0.19			
**Gender**				0.59	0.441
Male	1,240	47.38	9.00 (7.00-10.00)		
Female	1,369	52.31	9.00 (7.00-10.00)		
Missing value	8	0.31			
**Additional degrees to MD**				12.35	*P* < 0.001
No additional degree	291	11.21	8.00 (5.00-10.00)		
Bachelor	1,350	51.59	9.00 (7.00-10.00)		
Master	692	26.44	9.00 (7.00-10.00)		
Doctor	277	10.58	9.00 (7.00-10.25)		
Missing value	7	0.27			
**Marital status**				2	0.136
Single	397	15.17	9.00 (7.00-10.00)		
Married	2,148	82.08	9.00 (7.00-10.00)		
Divorced or Widowed	70	2.67	10.00 (8.00-10.00)		
Missing value	2	0.08			
**Professional position**				14.13	*P* < 0.001
Without professional title	306	11.69	8.00 (5.00-10.00)		
Resident doctor	564	21.55	9.00 (7.00-10.00)		
Attending physician	898	34.31	10.00 (7.00-11.00)		
Associate chief physician	569	21.74	9.00 (7.00-11.00)		
Chief Physician	270	10.32	9.00 (6.00-10.00)		
Missing value	10	0.38			
**Shift work**				62.13	*P* < 0.001
Dayworker	503	19.22	8.00 (5.00-10.00)		
Dayworker, but occasional work at night	500	19.11	8.50 (5.00-10.00)		
Night shift	1,613	61.64	8.67 (8.00-11.00)		
Missing value	1	0.04			
**Working time (hours)**				74.43	*P* < 0.001
≤ 8	528	20.18	7.00 (4.00-10.00)		
9-10	1,297	49.56	9.00 (7.00-10.00)		
11-12	504	19.26	10.00 (8.00-10.00)		
≥13	285	10.89	10.00 (9.00-11.00)		
Missing value	3	0.11			
**Psychological detachment**				55.12	*P* < 0.001
None detachment	1,514	57.85	10.00 (8.00-11.00)		
Little detachment	781	29.84	8.00 (6.00-10.00)		
Partly detachment	102	3.90	8.00 (5.25-10.00)		
Most detachment	182	6.95	7.00 (3.00-10.00)		
Overall detachment	35	1.34	8.00 (5.00-10.00)		
Missing value	3	0.11			
**Continuous working under status of sickness**				100.21	*P* < 0.001
Rest by organization's suggestion	39	1.49	5.00 (3.75-7.25)		
Rest by self-decision	169	6.46	6.00 (2.00-9.00)		
Generally, to continue working	1,831	69.97	9.00 (7.00-10.00)		
To continuous working by force	578	22.09	10.00 (9.00-11.00)		

Of the 2,617 doctors surveyed, most (66.6%) of the participants were from tertiary hospitals. The age of participants ranged from 20 to 53 years. Females made up 52.3% of the sample. Most (82.1%) participants were married. As for educational background, 51.7% obtained bachelor's degrees and 38.1% obtained master's or doctorate degrees. A total of 306 (11.7%) participants were without professional titles, 564 (21.6%) resident doctors, 898 (34.4%) attending physicians, 569 (21.8%) associate chief physicians, and 270 (10.4%) chief physicians. The representativeness of the responding doctors was evaluated by comparing characteristics of the study participants with those of Chinese doctors which were published in Chinese Health and Family Planning Statistical Yearbooks (CHFPSY), regarding age groups distribution (22.40% under 30 years in study participants vs. 22.10% in the CHFPSY report), gender (47.40 vs. 55.90% males), service years (16.50% under 30 service years in study participants vs. 23.40% in the CHFPSY report) and education level (44.30 vs. 51.70% bachelor in the CHFPSY report). Thus, sampling bias is also potential interference factor, resulting in an overreported doctors' dissatisfaction and/or those who complain of their professional situation.

A total of 2,617 (86.8%) respondents reported their NFR status. The median score of the overall level of NFR of Chinese doctors was nine points (range = 0-11). This result indicates that Chinese doctors have severe work-related fatigue.

As shown in Table 1, the differences in NFR scores for gender and marital status were not statistically significant (*P* > 0.05). With respect to age, the highest NFR was found among participants aged 31-40 years (*P* < 0.001). Length of service between 11 and 20 years had higher NFR scores than groups of other service years (*P* < 0.001). Doctors working in the large-scale general hospitals had higher levels of NFR than those in low-level hospitals. Doctors with master's and doctoral degrees reported significantly higher NFR than all others (*P* < 0.001) and attending physicians had higher NFR scores than all others (*P* < 0.001). Approximately 79.8% of participants reported working more than 8 h a day. Over one-half (61.6%) of participants who often worked at night had higher NFR scores than those with less night duty (*P* < 0.001). Respondents reporting prolonged working times had higher levels of NFR (*P* < 0.001). Most of the doctors reported low levels of psychological detachment (no detachment = 57.9%, little detachment = 29.8%); they also had higher NFR scores (*P* < 0.001). Approximately 70.0% of participants reported they generally continued to work when they are ill, and 22.1% of participants must continue to work long hours; they reported higher NFR scores than those who choose to rest (*P* < 0.001).

### Information on and Distribution of all Variables

The means, standard deviations, medians, and IQRs are provided in Table 2.

**Table 2:** The information of distribution of each variable.

**Variables**	**Mean**	**SD**	**Median**	**IQR**
1. Need for recovery	8.17	2.94	9	1-11
2. Life satisfaction	2.48	1.03	3	1-5
3. Psychological stress	3.33	0.86	3	1-5
4. Subjective career success	2.87	0.89	3	1-5
5. Subjective sleep quality	2.29	0.76	2	1-4
6. Subjective health	2.47	0.64	3	1-4

*SD, standard deviation; IQR, inter-quartile range*.

### Correlations Between Study Variables

Pearson correlation coefficients between the continuous variables are shown in Table 3. As indicated in the table, all variables were significantly correlated with each other. NFR was positively correlated with psychological stress (*r* = 0.470, *P* < 0.001), and negatively correlated with life satisfaction (*r* = −0.409, *P* < 0.001), career success (*r* = −0.245, *P* < 0.001), subjective sleep quality (*r* = −0.398, *P* < 0.001) and subjective health (*r* = −0.428, *P* < 0.001).

**Table 3:** Means, standard deviations (SD), and correlations of continuous variables.

**Variables**	**1**	**2**	**3**	**4**	**5**	**6**
1. Need for recovery	1					
2. Life satisfaction	−0.409^**^	1				
3. Psychological stress	0.470^**^	−0.389^**^	1			
4. Subjective career success	−0.245^**^	0.352^**^	−0.223^**^	1		
5. Subjective sleep quality	−0.398^**^	0.350^**^	−0.471^**^	0.227^**^	1	
6. Subjective health	−0.428^**^	0.379^**^	−0.464^**^	0.278^**^	0.524^**^	1

** *P < 0.001*.

### Hierarchical Linear Regression Models

Two hierarchical regression analyses were performed to examine the influence of NFR on workplace well-being (Table 4) and self-reported health outcomes (Table 5) after controlling for the effects of the demographic variables. Need for recovery was posed as a independent variable in this study, and workplace well-being (life satisfaction, psychological stress, and career success) and self-reported health outcomes (subjective sleep quality and subjective health) as dependent variables, respectively. Step 1 explained the influence of demographic variables (age, service years, hospital level, education level, professional title, shift work, working time, psychological detachment, and continuous working under sickness status) on each dependent variable. Step 2 explained joint influence of demographic variables and explanatory variable on response variable.

**Table 4:** Hierarchical linear regression models for workplace well-being.

**Variables**	**Life satisfaction**		**Psychological stress**		**Career success**	
	**Step1(β)**	**Step2(β)**	**Step1(β)**	**Step2(β)**	**Step1(β)**	**Step2(β)**
**Control variables**						
Age	0.076	0.074	0.031	0.033	−0.019	−0.020
Service Years	0.009	−0.003	0.014	0.031	0.120^*^	0.110
Hospital level	−0.007	−0.017	−0.008	0.003	0.008	0.002
Education level	0.041	0.043	0.009	0.007	0.050	0.051^*^
Professional title	−0.104^***^	−0.090^**^	0.018	−0.001	0.128^***^	0.139^***^
Shift work	−0.090^***^	−0.064^**^	0.032	0.000	−0.100^***^	−0.081^***^
Working time	−0.117^***^	−0.074^**^	0.157^***^	0.103^***^	−0.044	−0.012
Psychological detachment	−0.085^***^	−0.038	0.182^***^	0.120^***^	0.050^*^	0.086^***^
Continuous working under sickness status	−0.277^***^	−0.210^***^	0.224^***^	0.138^***^	−0.135^***^	−0.085^***^
**Need for recovery**		−0.293^***^		0.374^***^		−0.219^***^
*F*	44.666^***^	62.933^***^	45.257^***^	80.312^***^	27.370^***^	35.349^***^
ΔR^2^		0.070^***^		0.115^***^		0.039^***^

* *P < 0.05.*

** *P < 0.01.*

*** *P < 0.001 (two-tailed)*.

**Table 5:** Hierarchical linear regression models for self-reported health outcomes.

**Variables**	**Subjective sleep quality**		**Subjective health**	
	**Step1(β)**	**Step2(β)**	**Step1(β)**	**Step2(β)**
Age	0.054	0.053	−0.050	−0.052
Service years	−0.083	−0.099	0.008	−0.009
Hospital level	−0.009	−0.019	0.017	0.006
Education level	0.083^***^	0.085^***^	0.069^**^	0.071^**^
Professional title	0.009	0.026	−0.027	−0.008
Shift work	−0.064^**^	−0.036	−0.047	−0.015
Working time	−0.165^***^	−0.117^***^	−0.139^***^	−0.086^***^
Psychological detachment	−0.087^***^	−0.033	−0.085^***^	−0.025
Continuous working under sickness status	−0.185^***^	−0.109^***^	−0.189^***^	−0.104^***^
**Need for recovery**		−0.332^***^		−0.370^***^
*F*	29.452^***^	53.123^***^	25.049^***^	55.390^***^
ΔR^2^		0.091^***^		0.113^***^

*p < 0.05.

** *p < 0.01.*

*** *p < 0.001 (two-tailed)*.

As shown in Table 4, NFR accounted for an additional 7.0% of the variance in the prediction of life satisfaction in step 2. The test of Δ*R*^2^ was significant, indicating that NFR was a significant predictor of life satisfaction. NFR was negatively associated with life satisfaction (β = −0.293, *P* < 0.001). A positive correlation between NFR and psychological stress was observed in our respondents (β = 0.374, *P* < 0.001). We found that NFR was negatively associated with career success (β = −0.219, *P* < 0.001). In consideration of all of the above-mentioned factors, these results suggest NFR had a negatively association with the workplace well-being of Chinese doctors.

As indicated in Table 5, NFR accounted for an additional 9.1% of the variance in the prediction of subjective sleep quality in step 2. The test of Δ*R*^2^ was significant. Thus, these data are consistent with the notion that NFR is a significant predictor of subjective sleep quality (β = −0.332, *P* < 0.001). Consistent with the above results, NFR was also negatively associated with subjective health (β = −0.370, *P* < 0.001). Overall, these two findings demonstrated that NFR also had a negative association with doctors' self-reported health outcomes.

## Discussions

In the present study, a cross-sectional survey was conducted to examine the extent of NFR of Chinese doctors. This study also investigated whether NFR differs in terms of doctors' demographic variables. Another purpose of this study was to examine the relationship between NFR, workplace well-being, and subjective health outcomes. These cross-sectional data demonstrated that the median score of the overall level of NFR of Chinese doctors was nine points (range = 0-11). Given that the maximum score on this scale is 11, this indicates that the respondents experienced a high level of NFR. There are no data available in previous studies providing the distribution of NFR scores in the general population, which makes it difficult to determine if the level of NFR among Chinese doctors is comparatively high. However, the results still indicated that participants exhibited serious work-induced fatigue without being adequately recovered, as the findings indicated that almost two-thirds (61.6%) of the participants often work during the night and 79.8% of them needed to work more than 8 h every day; 10.9% of participants even reported they had to work more than 13 h every day. As a consequence, the workloads of Chinese doctors are very heavy (3), which tend to cause tiredness and even sudden death for some doctors (8). Previous research has suggested that most doctors object to their next generation working in the medical industry (49).

The present study provided further insight into work-induced fatigue and NFR of Chinese doctors. This study's results demonstrated that the highest NFR was found among attending physicians, doctors aged 31-40 years, and those with 11-20 years of work experience; these findings are consistent with previous studies (19) indicating that increasing age is associated with NFR. However, there is a further explanation for this result. In China, attending physicians aged 31-40 with 11-20 years work experience undertake more work roles and extreme work overloads (5). They often take more on night duty and have less time for rest and leisure; along with increasing higher psychosocial job demands, these factors all contribute to their fatigue.

The data in this study illustrated that higher educational and hospital levels are associated with higher NFR. Tertiary hospitals and high educational levels are scarce resources in China. It is understandable that doctors with higher educational levels in tertiary hospitals are the busiest. To provide quality healthcare services to a large number of patients (3), they are limited in opportunities for rest time. This is also a reflection of an insufficient quantity of high quality medical resources in China. The population of China is huge, which means that the medical needs of patients are very large as well. High-level hospitals solve this problem by increasing service quantity and the workload of doctors. In this case, better doctors become busier, which leads to the fact that their risks of fatigue and health burdens also become higher. Shift work as an important predictor for NFR was verified in our study; a similar result was observed by Maastricht Cohort Study (50). Moreover, another study among physicians reported that shift work of emergency physicians can lead to disturbed sleep and increased fatigue and neurohormonal results (51). Although the negative health effects of on-call work have been demonstrated in previous research (52), our investigation illustrated that 61.6% of participants were often working during the night in China. This means that they likely have inadequate time to recuperate from work-related efforts because of their on-call duties, which was also an important reason for their fatigue. Results from Tables 4, 5 also indicate that doctors who engage in longer work times are more likely to experience low life satisfaction, high levels of psychological stress, and poor sleep quality and health. Likewise, the present study also found that prolonged work hours are associated with high levels of NFR. This conclusion is strikingly similar to HÄRMÄ's (53), who found that doctors who must deal with prolonged or repeated exposure to stress, often experience a sustained arousal without the opportunity for adequate recovery (53). High job demands may result in doctors being unable to control their duties (54), which in turn increases the risk of negative health-related outcomes (33). Our regression results also suggest that shift work is more likely to threaten doctors' perceived life satisfaction and subjective career success, and even trigger sleep problems. In addition, the present findings also suggest that for many doctors it was very difficult to attain psychological detachment from work during off-job time (no detachment was 57.9%, little detachment was 29.8%), which suggests that doctors were still occupied by work-related duties during off-job time, in turn leading to higher NFR. As a partial mediator between job stressors and low work-home boundaries on the one hand and strain reactions (emotional exhaustion, need for recovery) on the other hand (24), psychological detachment refers to a specific cognitive–emotional state in which individuals mentally disconnect or psychologically detach from work and do not need to deal with job-related issues during their off-job time (55). Thus, insufficient psychological detachment is a potential explanatory mechanism for the relationship between NFR and workplace well-being (24). The stressor-detachment model suggests that work stressors from doctors' duties or demands are a serious impediment to the psychological detachment of doctors from work during non-work time, which will further cause doctors' strain reactions and negatively impact affective states, psychological reactions, and workplace well-being (56). Additionally, the results of the two regressions (Tables 4, 5) also indicate that a doctor with a state of psychological detachment during non-work time is likely to experience better workplace well-being and self-reported health outcomes. Additionally, when doctors were uncomfortable or experiencing illness, 70.0% of participants reported they generally continued working, and 22.1% of participants must continue working under the policies of the organization, resulting in higher NFR scores than those who are able to rest. This finding indicates that not only psychological detachment of doctors was difficult to obtain in non-working time, but also doctors' bodies can't get rest even when they are ill. This has seriously affected the interests, well-being, and health-related outcomes of doctors. A considerable amount of research has been conducted on many careers during the last decade, but few have been conducted with doctors. This study found that doctors with elevated NFR had lower workplace well-being (57) and health status (26), and that this was closely related to their quality of life. These results are similar to previous studies (58), but those findings were reported in professional fields other than medicine. In fact, other studies have demonstrated that NFR plays an important mediating role on the relationship between overtime or overload and health of employees (31, 57). High workload combined with lack of control (59), together with unreasonable work arrangements, reduces the recovery opportunity of doctors, resulting in the body and mind experiencing long-term NFR (31), so their well-being and health are adversely affected. The cognitive activation theory of stress (60) provides more insights and explanations of this association. Overtime work and excessive workload easily lead to sustained activation, combined with insufficient recovery from work. This can result in negative load consequences, followed by damaged individual well-being and health. Relevant pathophysiological mechanisms were also reported in previous findings (61). Overall, the main contribution of our study is to highlight that there is considerable NFR among Chinese doctors, causing considerable of work-induced fatigue. In China, doctors face a heavy workload, prolonged work times, frequent shift work, lack psychological detachment, and even have no choice but to continue working when they are ill, all of which result in reducing the work life quality of doctors. These are all considerable risks for elevated NFR, which in turn will damage the interests, well-being, and health-related outcomes of doctors. These effects even spread to medical service results (3). Corresponding interventions and polices for reducing work-induced fatigue have been introduced in many developed regions such as Australia, New Zealand, Europe, and the USA (62). Unfortunately, these issues aren't on the agenda in China. It remains poorly understood that these factors are of great importance for doctors' individual well-being and the country's health-care system. In this study, five suggestions were put forward from three aspects of individual doctors, hospitals and institutions to relieve the fatigue of doctors in tertiary hospitals who work for a long time. First of all, doctors should develop hobbies outside work. We encourage doctors to spend more time on sports, leisure and entertainment to relieve work pressure. Second, we suggest that doctors use mindfulness to adjust their inner state and timely recover their mind. Third, it is suggested that the hospital optimize the shift system in order to allow doctors in the tertiary hospitals to get sufficient rest. Fourthly, we suggest that the hospital organize medical staff to carry out “employee assistance plan” to help each other to reduce the pressure of life. Fifthly, the hierarchical diagnosis and treatment system needs to be continuously promoted in order to reduce the workload of doctors in tertiary hospitals. It is earnestly hoped that this study can motivate some policy makers in China to address doctors' occupational health and well-being, as well as lead to the development of specific targets for prevention of fatigue and high NFR.

## Limitations

Although there are significant findings in the present study, the study has several limitations. First, a convenience sample was used in this study, which raises the potential for sampling bias resulting in a non-random sample of a population in which all individuals were not equally likely to have been selected. Thus, we cannot provide complete confidence in the results of the overall investigation. Second, a cross-sectional design prevented the determination of causation related to the relationship between the NFR, workplace well-being, and health; thus, one important direction is that longitudinal studies should be conducted in the future. Third, the data were collected from self-reports of doctors, which introduces the possibility of response bias from social desirability or negative affect. The doctors might have overestimated or underestimated the association between the study variables. Moreover, using online questionnaires in an unmonitored setting is more likely to result in those experiencing difficulties responding. Further, it is not known whether those returning incomplete questionnaires differed in any significant way from those who completed them.

## Conclusions

To our knowledge, this is the first investigation to measure the prevalence and status of the NFR and associated factors among Chinese doctors, which seems to be often ignored although it is a very important dimension. This paper also indicated that the need for recovery is significantly related to workplace well-being and subjective health of doctors. The present study also provides insight into work overload, psychological detachment, and continuous working in spite of illness of Chinese doctors, which suggests some new perspectives for future research. In summary, we have identified that Chinese doctors are caught in a pattern of inadequate recovery outside work and are suffering considerable work-induced fatigue; their interests and well-being are facing serious challenges, and their health is threatened. The results in the present study suggest that some necessary actions should be implemented to put an end to the spreading crisis.

## Data Availability Statement

The raw data supporting the conclusions of this article will be made available by the authors, without undue reservation.

## Ethics Statement

Written informed consent was obtained from the individual(s) for the publication of any potentially identifiable images or data included in this article.

## Author Contributions

TS conceived, designed, and performed the experiments and wrote the paper. TS, YS, DY, and SZ analyzed the data. TS, YS, DY, and XW contributed reagents, materials and analysis tools. TS and XW approved the final manuscript for publication. All authors contributed to the article and approved the submitted version.

## Conflict of Interest

The authors declare that the research was conducted in the absence of any commercial or financial relationships that could be construed as a potential conflict of interest.

## Abbreviations

ECHMU: Ethics Committee of the Harbin Medical University
NFR: need for recovery
NFRS: need for recovery scale.
